# Elevated Homocysteine Level and Folate Deficiency Associated with Increased Overall Risk of Carcinogenesis: Meta-Analysis of 83 Case-Control Studies Involving 35,758 Individuals

**DOI:** 10.1371/journal.pone.0123423

**Published:** 2015-05-18

**Authors:** Donghong Zhang, Xuemei Wen, Wei Wu, Ye Guo, Wei Cui

**Affiliations:** 1 Department of Clinical Laboratory, Peking Union Medical College Hospital, Peking Union Medical College and Chinese Academy of Medical Sciences, Beijing, China; 2 Department of Clinical Laboratory, Fudan University Cancer Center, Shanghai, China; Duke Cancer Institute, UNITED STATES

## Abstract

**Background:**

Results of the association of folate metabolism and carcinogenesis are conflicting. We performed a meta-analysis to examine the effect of the interaction of serum concentration of homocysteine (Hcy), folate, and vitamin B12 and 5,10-methylenetetrahydrofolate reductase (MTHFR) polymorphism on risk of cancer overall.

**Method:**

Two reviewers independently searched for all published studies of Hcy and cancer in PubMed, EMBASE-MEDLINE and Chinese databases. Pooled results were reported as odds ratios (ORs) and mean differences and presented with 95% confidence intervals (95% CIs) and 2-sided probability values.

**Results:**

We identified 83 eligible studies of 15,046 cases and 20,712 controls. High level of Hcy but low level of folate was associated with risk of cancer overall, with little effect by type of cancer or ethnicity. Vitamin B12 level was inversely associated with only urinary-system and gastrointestinal carcinomas and for Asian and Middle Eastern patients. As well, MTHFR C677T, A1298C and G1793A polymorphisms were related to elevated serum level of Hcy, and folate and vitamin B12 deficiency. However, only MTHFR C677T homogeneity/wild-type (TT/CC) polymorphism was positively associated with overall risk of cancer.

**Conclusion:**

Elevated serum Hcy level and folate deficiency are associated with increased overall risk of cancer.

## Introduction

**Fig 1:**
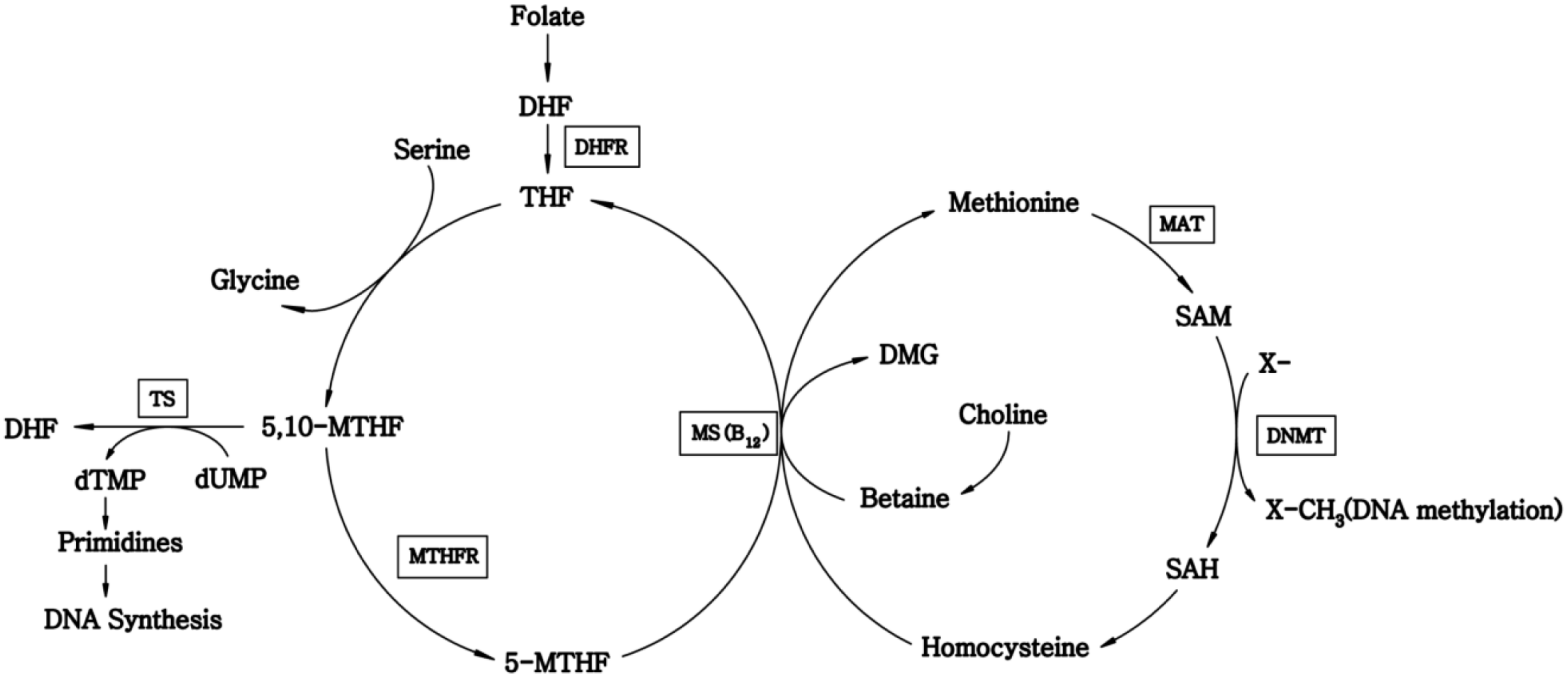
Flow diagram of homocysteine metabolism. MAT: methionine adenosyl transferase; SAM: S-adenosyl-methionine; DNMT: DNA methyltransferase; SAH: S-adenosylhomocysteine; MS: methionine synthase; DMG: dimethylglycine; THF: tetrahydrofolate; DHFR: dihydrofolate reductase; DHF: dihydrofolate; 5,10-MTHF: 5,10-methylenetetrahydrofolate; MTHFR: methylenetetrahydrofolate reductase; 5-MTHF: 5-methyltetrahydrofolate; TS: thymidylate synthase; dTMP: deoxythymidine monophosphate; dUMP: deoxyuridine monophosphate;

Cancer is the leading cause of death in economically developed countries and the second leading cause of death in developing countries [1]. Understanding the risk factors of cancer may guide the development of strategies targeting its prevention. Nutrigenomics focuses on the interactions between nutritional and genetic factors linked to risk of cancer [2]. Of note, elevated serum concentration of total homocysteine (Hcy), a well-known cardiovascular risk factor, and consequent deficiency of folate, vitamin B12, or vitamin B6, or genetic polymorphisms involves the transfer of one-carbon groups. The mechanism has been considered critical for Hcy metabolism in carcinogenesis in terms of DNA synthesis, repair and methylation (Fig 1) [3,4]. However, the underlying mechanism remains elusive.

Folate may play a significant role in determining the risk of developing several human epithelial cell malignancies, including cancer of the breast, ovary, pancreas, brain, lung, prostate and cervix, although the evidence is not consistent [5–8]. Despite evidence of an association of pathophysiologic folate status and cancer, epidemiologic data regarding folate supplementation from most human studies (retrospective, case—control, prospective and meta-analyses) are still conflicting, with some studies showing decreased risk[9,10] and others reporting no association[11] or even increased risk[12]. Similar data were found for Hcy and vitamin B6 status [6,13,14].

Some genetic studies suggest that 5,10-methylenetetrahydrofolate reductase (MTHFR) genetic polymorphisms related to serum Hcy and folate status can further affect cancer risk [15]. Of note, MTHFR C677T (rs1801133) and A1298C (rs1801131) polymorphisms have been found associated with reduced enzyme activity and increased serum level of total Hcy (tHcy), mostly in patients with folate deficiency. The effect of MTHFR polymorphism on cancer and pre-cancer risk remains controversial in terms of cancer site and the combined effects of other risk factors. The polymorphisms have a protective effect on colorectal[16] and gastric cancer[17] but a negative effect on prostate cancer[18] and no effect on head and neck or lung cancer[19]. These conflicting results are probably due to the relatively small number of cases studied, the detection method used and the genetic heterogeneity of the study populations, which disallows comparison of study results. First, studies have shown that genetic polymorphisms are affected by ethnicity and geographical differences. Scientists widely acknowledge that these two factors support the disparities for populations and are intensified by region in terms of understanding risk and response to drug treatment regimes. Second, several different detection methods for genetic polymorphisms include PCR-RFLP, allele-specific PCR, and DNA chip. Different methods with different sensitivities and accuracies might affect the results.

Therefore, we aimed to determine the possible association of serum concentrations of Hcy, folate, and vitamin B12 as well as MTHFR polymorphism and overall risk of cancer.

## Materials and Methods

### Study Search Strategy

We searched for all published studies evaluating the role of Hcy and its related metabolizing factors folate and vitamin B12 as well as MTHFR genotypes in cancer. Two investigators (X Wen, D Zhang) independently searched MEDLINE via PubMed, EMBASE and Chinese databases (Wanfang Data and CNKI Database) with the key words (“homocysteine”) AND (“cancer” OR “tumor” OR “carcinoma”) for articles in any language describing the association of serum Hcy concentration and cancer (up to November 2013). We also manually searched the reference lists of review and research articles for articles. All disagreements were resolved by discussion and with a third reviewer, if necessary.

Studies were included if they met the following criteria: (1) case—control studies; (2) diagnosis of cancers by immunohistochemical methods; (3) sufficient data for serum Hcy concentration (sample size, mean and SD) for both cases and controls; (4) subjects 18 years or older; and (5) containing an English abstract. If the same data were used in more than one publication, the latest or largest study was selected. The same 2 unmasked investigators independently assessed study validity. Quality was defined as (1) cancer patients without other complications such as diabetes, hypertension, myocardial infarction, stroke, renal failure, drug abuse or other serious diseases; (2) the control group age- and sex-matched to the case group; (3) cases not receiving any clinical or radiological anticancer treatment at the time of study; (4) cases and controls randomly selected; and (5) clear statement for the measurement of serum Hcy, folate and vitamin B12 levels as well as MTHFR genotyping, and the coefficient of variation for quality control repeats. Only studies that satisfied at least 3 criteria were defined as high-quality studies and were included. Agreement was analyzed by the kappa statistic, with Kappa >0.75 considered agreement between reviewers in study selection [20].

### Data Extraction

The following data were extracted from each eligible study: study design and execution; type of cancers; publication date; country of study; number of cases and controls; ethnicity; mean (SD) serum concentrations of Hcy, folate and vitamin B12; and MTHFR genotyping in cancer and controls.

### Statistical Analysis

The mean differences in serum concentrations of Hcy, folate and vitamin B12 in cases and controls were calculated by a random- or fixed-effects model to compute ORs with 95% CIs by the Z-test with use of RevMan 5.0. The heterogeneity of studies was evaluated by the Q-statistic at p<0.05. A random-effects model was used if heterogeneity was significant; otherwise, a fixed-effects model was applied. Subgroup analysis involved mainly cancer type, organ system and geographic location to explore potential sources of heterogeneity. The Z-test was used to calculate differences in serum concentrations of Hcy, folate and vitamin B12 by MTHFR genotype for cases and controls and for the association of the pooled OR for MTHFR polymorphism and risk of cancer. In our study, high Hcy concentration or deficiency of folate and B12 was evaluated relatively by comparing patients with cancer and controls.

Publication bias was assessed by funnel plots and statistically by Egger’s linear regression test with use of STATA 12.0. To be more conservative, the statistical significance level for interpreting Egger’s test results was p = 0.10. For cells with 0 values for number of events of interest, continuity correction was implemented by the addition of 0.5, whenever possible, to assess the presence of publication bias. To assess the stability of the meta-analysis results, one-way sensitivity analysis was performed by omitting each study one at a time from the analysis. Two-sided p<0.05 was considered statistically significant.

## Results

### Eligible Studies and Characteristics

**Fig 2:**
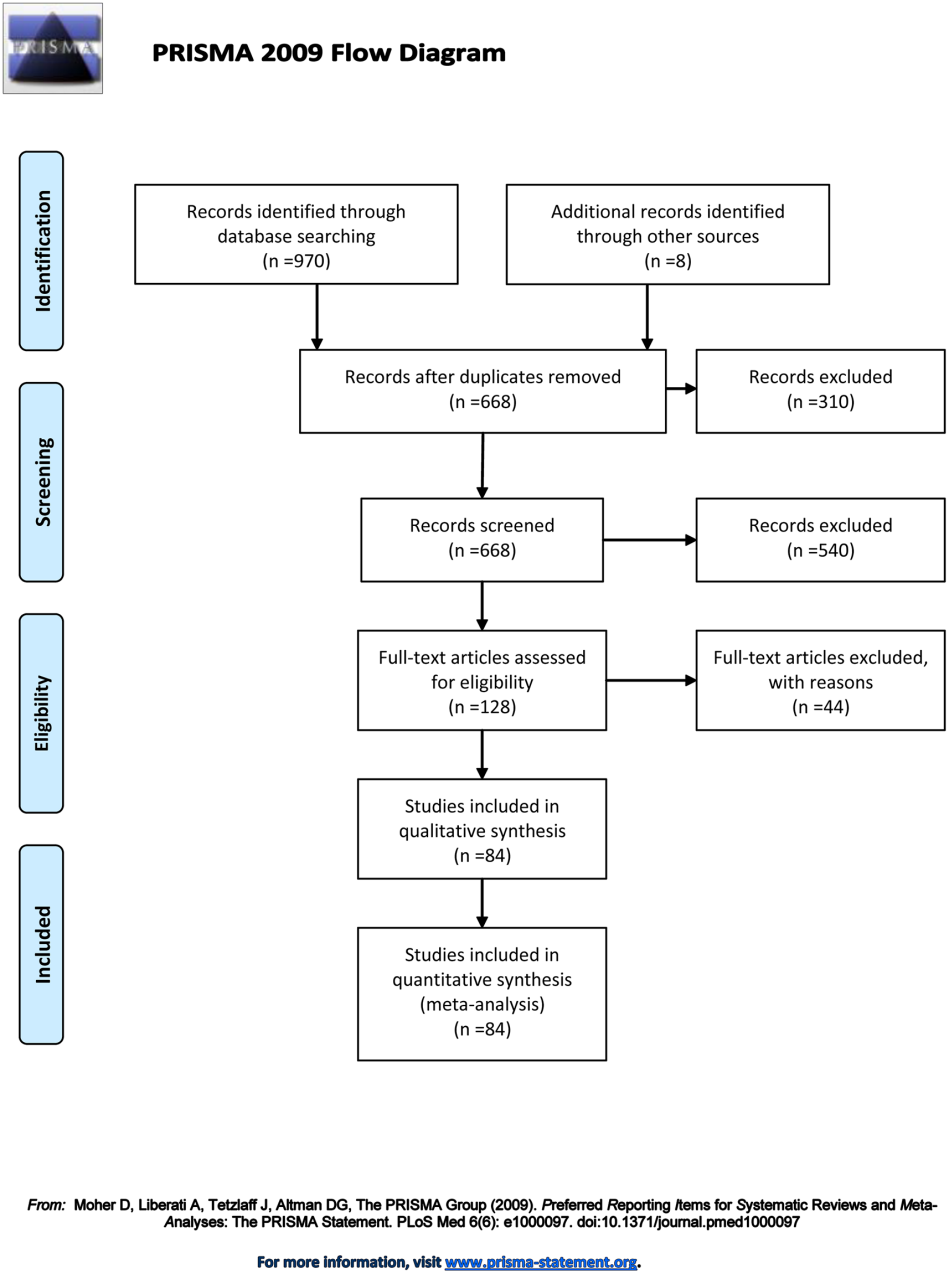
Flow diagram of the article selection for the meta-analyses.

In total, from 978 records searched, 83 eligible studies met our inclusion criteria (Fig 2). The characteristics of the studies are in Table 1. In all, 15,046 cases and 20,712 controls were investigated for Hcy; 40 studies [3,21–59] including 9,047 cases and 12,649 controls for folate; 28 studies [3,22,23,25,29–33,36,37,41–49,51–58] including 4,974 cases and 7,840 controls for vitamin B12; and 16 studies [3,26,27,38,42–45,50,60–66] including 5,657 cases and 6,557 controls for MTHFR C677T, A1298C, and G1793A polymorphisms. A total of 43 studies involved English-speaking subjects [3,21–25,27,28,30–50,60–73], 37 were performed in China [51–59,74–101], and 1 was performed in Korea [69], 1 in Brazil [102] and 1 in France [29]. The studies covered more than 14 types of cancer, including 22 [3,24,25,27,36,37,42,48,52,55,57,58,60,63,65,66,69,72,85,99–101] of colorectal cancer (CRC), 6 [46,52,55–96,101] of pancreatic cancer (PC), 11 [52,54,57,58,77–90,94,101,102] of esophagus cancer (EC), 16 [52,53,56–58,73,75,79,81,82,85,86,88,91,92,101] of hepatocellular carcinoma (HCC), 11 [52,57,58,76,79,80,83,85,90,91,101] of gastric cancer (GC), 16 [3,35,38,49,58,67,68,70,71,84,85,89–91,98,101] of breast cancer (BC), 4 [41,59,62,101] of cervical cancer (CC), 5 [59,74,91,93,101] of ovarian cancer (OC), 11 [23,32,34,40,51,85,87,91,95,97,101] of lung cancer (LC), 3 [21,22,79] of head and neck squamous cell carcinoma (HNSCC), 2 [31,39] of laryngeal squamous cell carcinoma (LSCC), 3 [30,43,58] of renal carcinoma (RCC), 5 [33,45,50,85,101] of prostate cancer (PCa), 2 [44,58] of bladder cancer (BLC) and 6 [26,29,47,61,64,101] of other cancers. In total, 11 of the articles [3,52,57–59,79,85,88,90,91,101] contained data for more than 2 different cancers, and we treated them independently in the analysis.

**Table 1:** Mean differences in homocysteine (Hcy), folate and vitamin B12 levels and cancer risk.

Cancer	Hcy				Folate				Vitamin B12			
	N^a^	Cases	Controls	OR (95% CI)	N^a^	Cases	Controls	OR (95% CI)	N^a^	Cases	Controls	OR (95% CI)
Total	83	15046	20712	**5.06** (4.59, 5.50)	40	9047	12649	**-2.68 (**-3.21, -2.15)	28	4974	7840	-18.52 (-47.17, 10.12)
**Subgroup by organ system**												
Digestive system	44	6198	8900	**5.90** (5.23, 6.57)	16	1823	3240	**-2.61 (**-2.98, -2.25)	14	1485	2577	**-31.14 (**-49.13, -13.15)
Genital system	24	5987	7233	**4.09** (3.05, 5.13)	10	4571	5060	**-1.65 (**-2.45, -0.85)	6	1285	1750	-28.57 (-104.64, 47.50)
Respiratory	13	1568	2648	**5.10** (2.86, 7.34)	7	1401	2343	**-2.11 (**-3.15, -1.07)	4	1293	2167	-35.58 (-89.83, 18.67)
Urinary system	5	569	599	1.01 (-0.20, 2.22)	5	724	1332	-2.09 (-5.15, 0.96)	4	574	1032	**-10.71 (**-16.36, -5.05)
Other	6	724	1332	**5.31** (1.35, 9.27)	5	528	674	**-2.48 (**-4.63, -0.33)	3	337	314	89.02 (-135.35, 313.39)
**Subgroup by cancer type**												
CRC	22	3954	5038	**2.63** (1.74, 3.51)	11	1181	1958	**-1.10 (**-1.60, -0.60)	10	873	1325	**-28.52 (**-50.60, -6.43)
PC	6	332	920	**3.97** (1.69, 6.24)	4	288	730	-4.46 (-9.46, 0.55)	4	288	730	**-3.04 (**-5.29, -0.80)
EC	11	302	663	**7.45** (5.05, 9.86)	4	107	169	**-6.04** (-8.63, -3.46)	4	107	169	2.48 (-52.73, 57.68)
HCC	16	846	911	**10.21** (7.72, 12.69)	5	152	224	**-4.60** (-5.93, -3.26)	4	122	194	**-97.53 (**-137.09, -57.96)
GC	11	717	1164	**8.01** (5.12–10.90)	3	95	159	**-4.67** (-6.66, -2.69)	3	95	159	-22.59 (-82.54, 37.36)
BC	16	2208	2475	**3.59** (2.35, 4.83)	5	1076	1123	-1.04 (-2.12, 0.05)	3	819	853	-14.70 (-52.02, 22.62)
CC	4	184	366	**4.81** (2.28, 7.34)	2	56	55	**-5.97** (-6.62, -5.32)	1	38	35	**-131.63 (**-132.40, -130.86)
OC	5	89	120	**7.56** (4.55, 10.57)	1	11	20	**-4.99** (-7.63, -2.35)				
LC	11	1483	2508	**4.73** (2.32, 7.14)	5	1316	2203	**-1.91** (-3.04, -0.78)	3	1233	2107	-25.04 (-85.66, 35.58)
HNSCC	3	216	445	6.29 (-0.12, 12.69)	3	335	570	**-3.97** (-4.62, -3.31)	1	144	210	-47.60 (-104.03, 8.83)
LSCC	2	85	140	7.81 (-3.33, 18.94)	2	85	140	**-2.42** (-4.72, -0.12)	1	60	60	**-66.55 (**-108.31, -24.79)
RCC	3	391	622	0.002 (-0.28, 0.29)	3	391	622	**-0.95** (-1.90, -0.003)	3	391	622	**-10.52 (**-17.53, -3.50)
PCa	5	3481	4082	2.51 (-1.54, 6.56)	3	3428	3862	0.13 (0.05, 0.21)	2	174	348	16.18 (-27.29, 59.64)
BLC	2	183	410	1.31 (-1.61, 4.23)	2	183	410	-1.46 (-4.68, 1.77)	2	183	410	**-11.06 (**-20.61, -1.51)
Others	6	575	848	**2.81** (1.48, 4.15)	3	343	404	-2.09 (-7.03, 2.85)	2	193	104	162.17 (-50.08, 374.42)
**Subgroup by location**												
Europe	21	6156	7067	**1.83** (1.34, 2.31)	15	4870	5822	**-1.17** (-1.55, -0.79)	9	1311	1714	-13.06 (-39.38, 13.28)
Asia	45	4064	6782	**6.87** (6.17, 7.57)	13	1157	1766	**-4.65** (-5.82, -3.47)	9	759	1195	**-43.25 (**-76.57, -9.92)
America	7	3165	3939	**0.33** (0.06, 0.59)	5	1387	2161	-0.24 (-0.54, 0.07)	4	1341	2117	-10.77 (-27.01, 5.46)
Middle East	6	626	1118	**2.64** (1.00, 4.278)	5	614	1114	**-1.40** (-2.40, -0.41)	4	544	1028	**-13.33 (**-23.87, -2.80)
Latin America	2	907	1767	3.41 (-2.18, 9.00)	1	891	1747	**-1.41** (-1.84, -0.98)	1	891	1747	12.70 (-12.31, 37.71)
Australia	1	128	39	**2.50** (1.88, 3.12)	1	128	39	0.40 (-0.05, 0.85)	1	128	39	264.00 (214.93, 313.07)

^a^ No. of studies.

OR, odds ratio; 95% CI, 95% confidence interval. CRC, colorectal cancer; PC, pancreatic cancer; EC, esophagus cancer; HCC, hepatocellular carcinoma; GC, gastric cancer; BC, breast cancer; CC, cervical cancer; OC, ovarian cancer; LC, lung cancer; HNSCC, head and neck squanlous cell carcinoma; LSCC, laryngeal squamous cell carcinoma; RCC, renal carcinoma; PCa, prostate cancer; BLC, bladder cancer.

### Association of Serum Levels of Hcy, Folate and Vitamin B12 and Cancer Risk

As compared with healthy controls, cancer risk was associated with high serum Hcy level (odds ratio [OR] 5.06; 95% confidence interval [95% CI] 4.59–5.52) but low folate level (-2.68; -3.21 to -2.15) (Table 1 and S1–S3 Figs), with no association for vitamin B12 (-18.52; -47.17 to 10.12).

We divided data for cancer patients into 5 groups by organ system: digestive-system carcinomas (including CRC, PC, EC, HCC, GC and cholangiocarcinoma), genital neoplasms (including CC, OC, PCa, BC and endometrial cancer), respiratory tumors (including LC and LSCC), urinary-system tumors (including RCC, BLC and one urothelial carcinoma) and other (including HNSCC, high-grade glioma and mixed tumor) (Table 1). Except for urinary-system tumors, risk of other system cancers was associated with elevated Hcy level and decreased folate level for patients as compared with controls. In contrast, vitamin B12 level was inversely associated with cancer risk for only urinary-system tumors (OR -10.71; 95% CI -16.36 to -5.05) and digestive-system carcinomas (-31.14; -49.13 to -13.15) as compared with controls.

We performed a further subgroup analysis based on type of cancer. Except for HNSCC, LSCC, RCC, PCa and BLC, risk of some cancers was associated with high Hcy level (OR 2.63 to 10.21) as compared with controls. Except for PC, BC, PCa, BLC and other cancers, risk of most cancers was inversely associated with folate level. Vitamin B12 level was inversely associated with risk of CRC (OR -28.52; 95% CI -50.60 to -6.43), PC (-3.04; -5.29 to -0.80), HCC (-97.53; -137.09 to -57.96), CC (-131.63; -132.40 to -130.86), LSCC (-66.55; -108.31 to -24.79), RCC (-10.52; -17.53 to -3.50) and BLC (-11.06; -20.61 to -1.51).

Given the typical diets of ethnic groups, we performed a subgroup analysis by location of the study. The cancer risk associated with high Hcy level was sustained in each geographic location except Latin America, but the protective effect of folate was found only in Europe (OR -1.17; 95% CI-1.55 to -0.79), Asia (-4.65; -5.82 to -3.47), the Middle East (-1.40; -2.40 to -0.41) and Latin America (-1.41; -1.84 to -0.98) and that of vitamin B12 only in Asia (-43.25; -76.57 to -9.92) and the Middle East (-13.33; -23.87 to -2.80).

### Association of MTHFR C677T, A1298C and G1793A Polymorphism and Risk of Cancer

MTHFR polymorphisms are associated with reduced enzyme activity and increased total Hcy (tHcy) level. We explored the association of MTHFR C677T, A1298C and G1793A polymorphisms and risk of cancer and found only MTHFR C677T homogeneity/wild-type (TT/CC) polymorphism positively associated with overall risk (OR 1.18 (95% 1.05–1.33), Table 2).

**Table 2:** Meta-analysis of the association of MTHFR polymorphism and overall cancer risk.

MTHFR Genotype	N^a^	Heterogeneity/Wild type			Homogeneity/Wild type			Heterogeneity+ Homogeneity/Wild type		
		Cancer	Control	OR (95% CI)	Cancer	Control	OR (95% CI)	Cancer	Control	OR (95% CI)
C677T	16	2171/2784	2600/3256	1.09 (0.95, 1.26)	702/2784	701/3256	**1.18 (1.05, 1.33)** ^b^	2873/2784	3301/3256	1.12 (0.98, 1.27)
A1298C	7	458/362	616/687	1.41 (0.92,2.17)	95/272	130/637	1.55 (0.91, 2.65)	451/272	685/637	1.57 (0.93, 2.64)
G1793T	3	39/445	74/894	1.06 (0.71, 1.59)						

^a^ No. of studies.

^**b**^ P<0.05.

### Heterogeneity Analysis and Publication Bias

We found heterogeneity for studies of Hcy, folate and vitamin B12 levels in the whole meta-analysis and on sub-group analysis. However, no further definite source of heterogeneity was identified. Egger’s test results suggested the absence of publication bias for levels of folate (P = 0.06) but not Hcy and vitamin B12 (P<0.001; P = 0.006, respectively) in cancer patients and controls, and the overall shape of the funnel plots revealed some asymmetry (S4 Fig). We found no significant publication bias for MTHFR C677T, A1298C and G1793A polymorphisms for overall risk of cancer, as suggested by funnel plots and Egger’s test results (P = 0.126 for C677T: CT/CC; P = 0.079 for C677T: CT+TT/CC; P = 0.474 for C677T:TT/CC; P = 0.950 for A1298C: AC/AA; P = 0.919 for A1298C: CC+AC/AA; P = 0.990 for A1298C: CC/AA; P = 0.383 for G1793T: GT/GG). Furthermore, no individual study predominantly affected the overall OR, because omission of any one study had no effect on results.

## Discussion

We performed a meta-analysis of studies involving 15,046 cases and 20,712 controls to examine the association of serum levels of Hcy as well as its metabolizing factors on overall risk of cancer. High serum level of Hcy and folate deficiency was consistently associated with risk of cancer, with little effect by type of cancer or ethnicity. However, only MTHFR C677T homogeneity/wild-type (TT/CC) polymorphism was positively associated with overall risk of cancer. Our study highlights the role of Hcy metabolism in carcinogenesis.

An association does not prove causality. Indeed, Hcy and folate have a critical role in maintaining DNA stability by donating one-carbon moieties [4,103]. Multiple studies have shown that folate deficiency induces epigenetic changes, which leads to global DNA hypomethylation, DNA repair, chromosomal instability, protooncogene activation, uracil misincorporation, DNA strand breakage, chromosomal breakage and malignant transformation. Moreover, hyperhomocysteinemia may promote inflammatory processes via oxidative stress [3,104], by increased levels of cell adhesion molecules, cytokines (interleukin 6 and tumor necrosis factor-α) and chemokines (high-sensitivity C-reactive protein), which may contribute to the biology of cancer. Thus, as for cardiovascular disease, supplementing folate to lower serum level of Hcy and repair DNA synthesis and methylation to prevent carcinogenesis might be reasonable. Recent findings from several large-scale human observational or placebo-controlled intervention trials indicated that supplementing synthetic folic acid decreased the risk of cancer at several sites, including the breast [105], colon [106], lung [107] and prostate [108]. However, data from animal models and human intervention trials suggested that supplementation with synthetic folic acid may enhance the induction of cancer cells[106,108,109]. In contrast, folate deficiency has an inhibitory effect on the progression of established neoplasms. Carcinogenesis is accelerated if folic acid is given after the emergence of lesions, presumably by providing DNA precursors for cancer cell growth [110]. Therefore, epidemiologic data from large-scale human trials is disappointing [106,111].

In our meta-analysis, we found an association of elevated serum Hcy level and deficent folate level and risk of cancer that differed by type of cancer, ethnicity and genetic polymorphisms. First, high serum Hcy level was associated with more than two-fold risk of each cancer type, and folate level showed an inverse association for each cancer type except PCa and BLC. Especially, the null association of levels of Hcy and folate and PCa risk was consistent with findings from Collin et al. [6]. Similar to Larsson et al. [112], we found no association of folate level and BC risk. Epidemiologic studies have consistently found moderate alcohol consumption associated with increased risk of BC [113,114], which may have influenced our findings in part.

Second, ethnicity is a significant contributor to the association of Hcy level and risk of cancer in each geographic location except Latin America, but the protective effect of folate was found only in Europe, Asia, the Middle East and Latin America and that of vitamin B12 only in Asia and the Middle East. Evidence of the ethnic effect of Hcy status mainly comes from South Africa. Black adults showed low Hcy concentrations and lacked the rightward skew of values seen for white adults [115]. Detailed study indicated that Mexican American females had significantly lower tHcy concentrations than non-Hispanic African-American females. As well, geometric mean tHcy concentrations were lowest in Mexican American girls [116]. However, black people did not show metabolic improvement after vitamin therapy as compared with white adults [117]. Limited research remains available about Hcy levels, but a significant association of the MTHFR 677C/T polymorphism is relatively common in white populations and is implicated in hyperhomocysteinemia [110]. Deficiency in vitamin B12 has been reported among vegetarians such as people in India, who cook vegetables for a longer period, drain the water from cooked vegetables, and rarely eat salads and raw vegetables[118]. Interestingly, in a Japanese cohort, vitamin B6 intake had a protective association with colorectal cancer only in men [119].

In addition, we found elevated Hcy level but not folate and vitamin B12 deficiency related to MTHFR C677T, G1793A and A1298C polymorphisms in controls or cases (data not shown). However, we found no significant positive association of MTHFR polymorphism (except for C677T homogeneity) and cancer risk. Similarly, MTHFR-knockout mice did not show increased incidence of tumor [120]. Furthermore, previous study found that the prevalence of the MTHFR 677T allele was 0.07 in sub-Saharan Africans and 0.06 in Canadian Inuit [121], so the impact of the MTHFR polymorphism on DNA methylation is likely minor in decreasing the susceptibility to cancer among Europeans or Americans. Thus, the association of MTHFR polymorphism and cancer risk is nonlinear. All these data might account for the inconsistent results for folate supplementation and cancer prevention.

The study’s main limitation is that blood samples were drawn after the occurrence of cancers. However, the status of Hcy, folate and vitamin B12 level over the long term is largely unknown, so causality cannot be directly inferred from our results. As well, we found only 83 relevant articles, for lack of sufficient data for an association of original or potential confounding factors such as diet and medication use, which further limited our evaluation of gene—environment interactions for cancer risk. Additionally, although some of the Egger tests and funnel plots showed some publication bias, a conservative p value was used and removal of each individual study did not significantly alter the results. Although about 44% of eligible studies were of Chinese people, we found no effect of Hcy, folate and vitamin B12 on risk of cancer in the total group or by subgroup in Asia when we deleted all Chinese. However, Chinese data highlighted the role of Hcy, folate and vitamin B12 in carcinogenesis. Finally, we evaluated heterogeneity in the whole and subgroup analyses. We explored several possible sources of heterogeneity, including ethnicity, sample size, quality score and control types but did not find a reason for this variation, so unknown confounding variables in single studies may have biased the findings. However, the quality of studies in our meta-analysis was satisfactory according our selection criteria, and we detected no publication bias, so pooled results were credible and stable.

In conclusion, the present meta-analysis is so far the largest study of an association of the circulating levels of folate-pathway vitamin and metabolite concentrations and overall risk of cancer, contributing 5.06- and 2.68-fold (inverse variance) by weight to the meta-analytical results for tHcy and folate levels, respectively. However, the type of cancer, ethnicity and genetic polymorphisms contribute to the effect of vitamin B12 on carcinogenesis. Supplementation with folate to prevent cancer should be individualized, taking into account diet, habits, folate status and MTHFR polymorphism.

## Supporting Information

S1 Fig Forest plot of serum Hcy levels for all cancers. (TIF)

S2 Fig Forest plot of serum folate levels for all cancers. (TIF)

S3 Fig Forest plot of serum vitamin B12 levels for all cancers. (TIF)

S4 Fig Begg’s funnel plot for Hcy, floate and vitamin B12 for all cancers. (TIF)
