# Supplementary figures and images for: Elevated Homocysteine Level and Folate Deficiency Associated with Increased Overall Risk of Carcinogenesis: Meta-Analysis of 83 Case-Control Studies Involving 35,758 Individuals
Source: PLoS One. 2015 May 18;10(5):e0123423. doi: 10.1371/journal.pone.0123423 (PMC4436268; doi:10.1371/journal.pone.0123423)

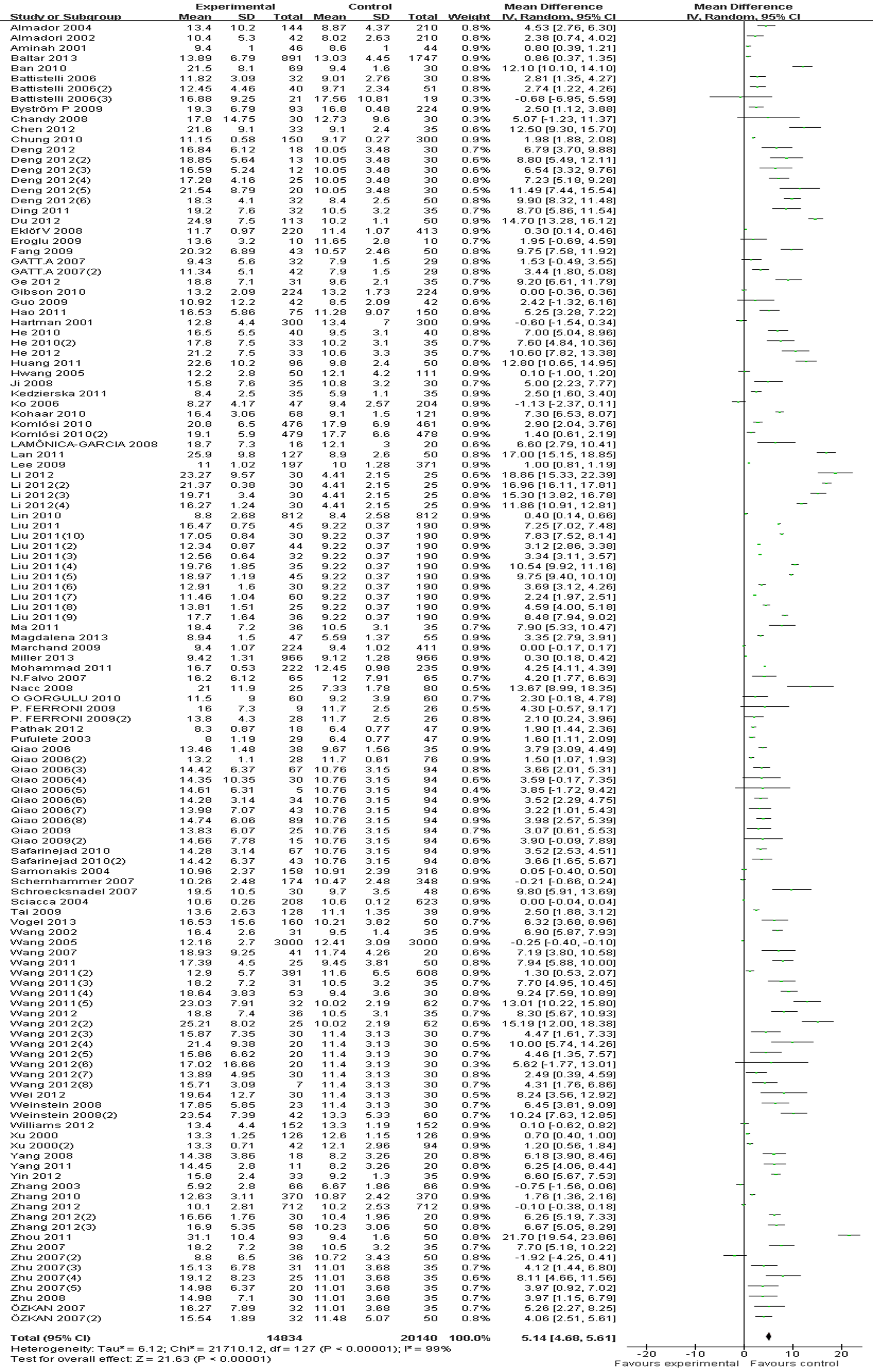

Supplement: S1 Fig — (TIF) [file pone.0123423.s001.tif]

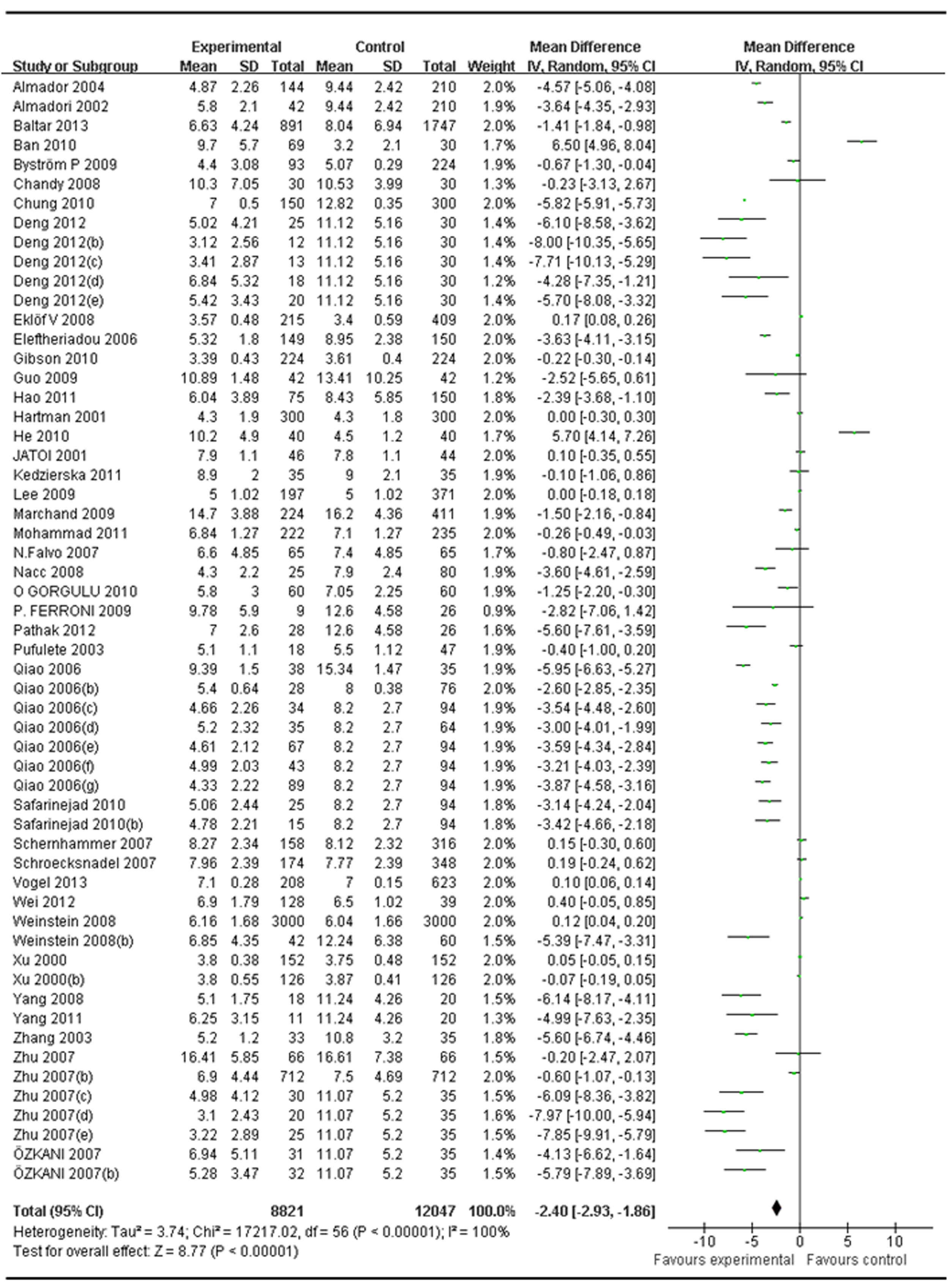

Supplement: S2 Fig — (TIF) [file pone.0123423.s002.tif]

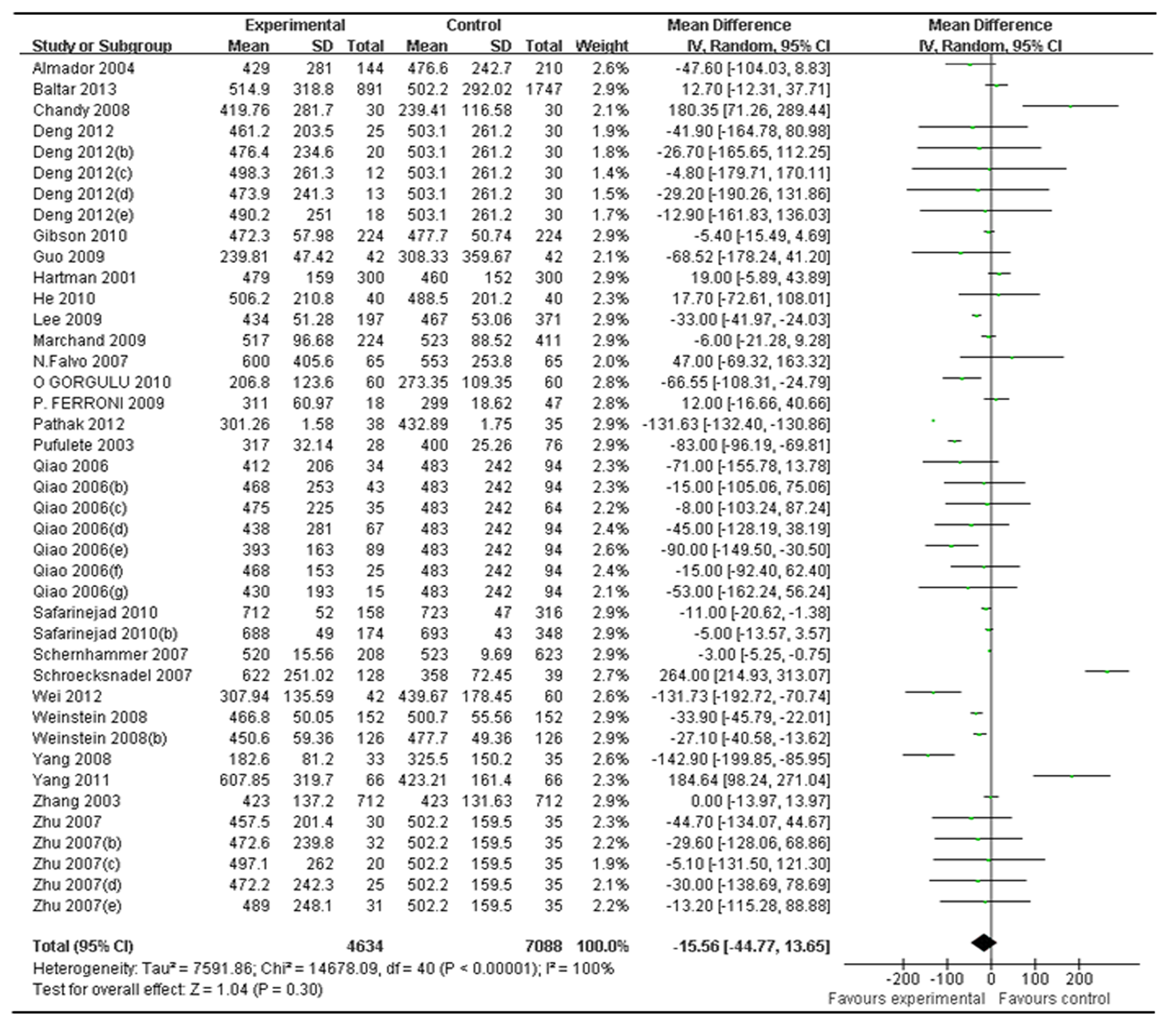

Supplement: S3 Fig — (TIF) [file pone.0123423.s003.tif]

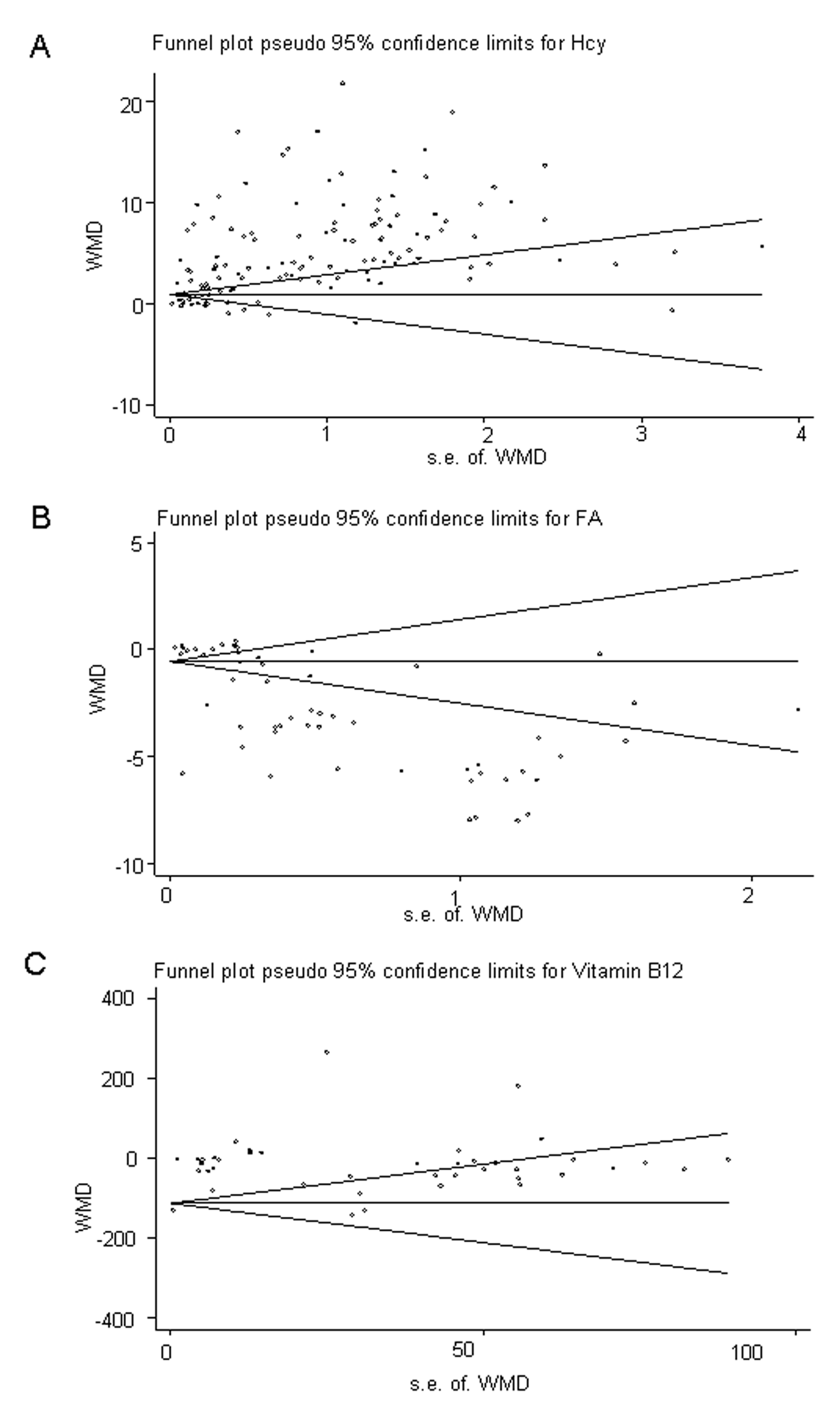

Supplement: S4 Fig — (TIF) [file pone.0123423.s004.tif]
